# Natural Products from Marine Fungi

**DOI:** 10.3390/md18050230

**Published:** 2020-04-27

**Authors:** Hee Jae Shin

**Affiliations:** 1 Marine Natural Products Chemistry Laboratory, Korea Institute of Ocean Science and Technology, 385 Haeyang-ro, Yeongdo-gu, Busan 49111, Korea; shinhj@kiost.ac.kr; 2 Department of Marine Biotechnology, University of Science and Technology (UST), 217 Gajungro, Yuseong-gu, Daejeon 34113, Korea

## Introduction

Marine fungi have been studied since the first record of the species *Sphaeria posidoniae* (*Halotthia posidoniae*) on the rhizome of the sea grass *Posidonia oceanica* by Durieu and Montagne in 1846 [1], but they have largely been neglected, even though it is estimated that there are greater than 10,000 marine fungal species. To date, a relatively small percentage of described species are associated with marine environments, with ~1100 species exclusively retrieved from the marine environment, although estimates for the number of fungal species on the planet range from 1.5 to over five million, likely fewer than 10% of fungi have been identified so far. Fungi have been found in nearly every marine habitat examined, including sediments, the water column, driftwood, sessile and mobile invertebrates, algae, and marine mammals, ranging in location from the deep sea all the way to surface waters [2].

It is believed that the exploration of marine fungi that are living in new and extreme habitats will advance the isolation of novel marine fungi and, thus, might lead to the isolation of novel secondary metabolites.

## Marine Fungal Metabolites

When considering that 38% of the approximately 22,000 bioactive microbial metabolites are of fungal origin, and that only about 5% of the world’s fungal taxa have been described, fungi exhibit a tremendous potential for the discovery of novel bioactive secondary metabolites [3]. Specifically, marine fungi still represent an underestimated but rich source for new secondary metabolites, although their distribution and ecological role often remain scarce. Marine fungi are an important source of secondary metabolites useful for the drug discovery. Even though marine fungi are less explored in comparison to their terrestrial counterparts, a number of useful hits have been obtained from the drug discovery perspective adding to their importance in the natural product discovery [4]. A number of metabolites from marine fungi have been discovered from various sources, which show a range of activities, such as antibacterial, antiviral, and anticancer agents. Although, over a thousand marine fungi related secondary metabolites have already been reported, except for cyclosporine A, none of them have reached the market yet, which could partly be related to non-comprehensive screening approaches and a lack of sustained lead optimization. Marine fungi are potent producers of polyketides, alkaloids, terpenes, peptides, and mixed biosynthesis compounds that represent chemical groups of secondary metabolites. 

In the review article by Zhao et al. (2018) [5], the novel natural products from extremophilic fungi were compiled. The authors focused on 314 novel compounds from 56 extremophilic fungal strains published from 2005 to 2017, highlighting the chemical structures and their biological potential. Extremophilic fungi have been found to develop unique defenses to survive extremes of pressure, temperature, salinity, desiccation, and pH, leading to the biosynthesis of novel natural products with diverse biological activities. This review focuses on the source, chemical structure types, biological activities, and references of all novel natural products and will help readers to better understand the underlying potential of fungal natural products as drug candidates. This review demonstrated that fungi from extreme environments are a rich source for novel natural products, even though the research on them is not as up-to-date as the research on fungi in other mesophilic environments due to the difficulties in both sample collection and cultivation.

Deep-sea fungi inhabit depths of thousand meters or below the surface where the sea environments are extreme, being typically characterized by the absence of sunlight irradiation, predominantly low temperature, high hydrostatic pressure, and oligotrophy [6]. Since the first report of fungal isolation from deep-sea [7], many fungi have been isolated from various deep-sea environments. Fungi isolated from the deep-sea samples are one of the most pivotal and promising source for bioactive compounds, presumably owing to the chemical diversity and biodiversity of their secondary metabolites that could be used for drug discovery and pharmacological applications [8]. 

From the deep-sea fungus *Phomopsis lithocarpus* isolated from a deep-sea sediment sample collected in the Indian Ocean at the depth of 3606 m, Zhang et al. isolated five new benzophenone derivatives, sharing a rare naturally occurring aldehyde functionality in this family, and a new eremophilane derivative, together with two known compounds [8]. One of the new compounds, tenellone H, exhibited cytotoxic activity against HepG-2 and A549 cell lines with IC_50_ values of 16.0 and 17.6 µM, respectively.

From another deep-sea fungal strain FL30r, identified as *Phialocephala* sp. obtained from a deep-sea sample (depth 5059 m), two new nitrogen-containing sorbicillinoids, named sorbicillasins A and B, and a new 3,4,6-trisubstituted α-pyrone derivative, scirpyrone K, together with two known biosynthetically related polyketides, were isolated by the OSMAC (one strain-many compounds) method [9]. Sorbicillasins A and B were unusual naturally occurring nitrogen-containing sorbicillinoid derivatives with a novel hexa-hydropyrimido[2, 1-*a*]isoindole moiety. Scirpyrone K exhibited radical scavenging activity against DPPH.

Symbiotic relationships are vast and diverse within the marine environment and many marine organisms, such as invertebrates as well as other marine macro-organisms, live in symbiosis with their microbial communities [10,11]. Marine sponges, sometimes referred to as microbial fermenters, are an outstanding source of highly diverse microbial communities, including new fungal species [12]. Sponge-derived fungi are one of the richest sources of many structurally unique and biologically active secondary metabolites among marine sources [13]. Seven new structurally diverse polyketide derivatives, along with 21 known compounds, were isolated from cultures of the sponge-derived fungus, *Alternaria* sp. SCSIO41014 by Liu et al. [14]. Altertoxin VII exhibited cytotoxic activities against human erythroleukemia (K562), human gastric carcinoma cells (SGC-7901), and hepatocellular carcinoma cells (BEL-7402), with IC_50_ values of 26.58 ± 0.80, 8.75 ± 0.13, and 13.11 ± 0.95 µg/mL, respectively. This compound is the first example possessing a novel 4,8-dihydroxy-substituted perylenequinone derivative, while the phenolic hydroxy groups have always commonly substituted at C-4 and C-9. In the work of Zu et al. [15], two novel aspochalasins, tricochalasin A, and aspochalasin A2, along with three known compounds, have been isolated from the different culture broth of *Aspergillus* sp., which was found in the gut of a marine isopod *Ligia oceanica* by employing the OSMAC approach by varying the culture conditions. Eight new fungal natural products, meroterpenoids and isocoumarinoids, were isolated from the culture of the salt-tolerant plant-associated fungus *Myrothecium* sp. OUCMDZ-2784 [16]. This study revealed that fungi living in the salt-tolerant plants are important biological resources for new and bioactive natural products.

Most of the studies on marine fungi have been made with those that are associated with marine sediments taken from shallow water or deep-sea and mangrove areas [17]. Five new anthraquinone derivatives, auxarthrols D–H, along with two known analogues, were isolated from the culture of the marine sediment-derived fungus *Sporendonema casei* [18]. Auxarthrols D and F showed cytotoxic activities, with IC_50_ values from 4.5 µM to 22.9 µM, while altersolanol B displayed potential antitubercular activity for the first time. In the experimental work of Song et al., six new diketopiperazines, three pairs of new brevianamides, were isolated from a marine-derived fungus strain *Aspergillus versicolor* MF180151 that was isolated from a sediment sample [19]. Shin et al. isolated six new phenalenone derivatives and five known compounds of the herqueinone class from a marine sediment-derived fungus *Penicillium* sp. [20]. 4-Hydroxysclerodin and an acetone adduct of a triketone exhibited moderate anti-angiogenetic and anti-inflammatory activities, respectively. A new alkenoic acid, fusaridioic acid A, three new bis-alkenoic acid esters, fusariumester A1, A2, and B, together with three known compounds, were isolated from the fungus *Fusarium solani* H915 derived from mangrove sediments by Qiu et al. [21]. Hymeglusin, an alkenoic acid derivative with a β-lactone ring, showed potent antifungal activity against tea pathogenic fungi with low toxicity.

In summary, the special issue “Natural Products from Marine Fungi” compiles the recent results from marine fungi. As a guest editor, I am grateful to all the authors who contributed their excellent results to the special issue, all the reviewers who carefully evaluated the submitted manuscripts, and the editorial boards of Marine Drugs, and Vincent Di, Assistant Editor, for their support and kind help.
